# She Thinks in English, But She Wants in Mandarin: Differences in Singaporean Bilingual English–Mandarin Maternal Mental-State-Talk

**DOI:** 10.3390/bs10070106

**Published:** 2020-06-27

**Authors:** Michelle Cheng, Peipei Setoh, Marc H. Bornstein, Gianluca Esposito

**Affiliations:** 1 Psychology, School of Social Sciences, Nanyang Technological University, Singapore 639818, Singapore; michellecheng@ntu.edu.sg (M.C.); gianluca.esposito@ntu.edu.sg (G.E.); 2 Eunice Kennedy Shriver National Institute of Child Health and Human Development, Bethesda, MD 20892, USA; marc.h.bornstein@gmail.com; 3 United Nations Children’s Fund, New York, NY 10017, USA; 4 Institute for Fiscal Studies, London WC1E 7AE, UK; 5 Lee Kong Chian School of Medicine, Nanyang Technological University, Singapore 636921, Singapore; 6 Department of Psychology and Cognitive Science, University of Trento, 38068 Rovereto, TN, Italy

**Keywords:** bilingualism, mental-state-talk, socialization

## Abstract

Chinese-speaking parents are believed to use less cognitive mental-state-talk than their English-speaking counterparts on account of their cultural goals in socializing their children to follow an interdependence script. Here, we investigated bilingual English–Mandarin Singaporean mothers who associate different functions for each language as prescribed by their government: English for school and Mandarin for in-group contexts. English and Mandarin maternal mental-state-talk from bilingual English–Mandarin mothers with their toddlers was examined. Mothers produced more ‘’cognitive’’ terms in English than in Mandarin and more ‘’desire’’ terms in Mandarin than in English. We show that mental-state-talk differs between bilingual parents’ languages, suggesting that mothers adjust their mental-state-talk to reflect the functions of each language.

## 1. Introduction

In toddlerhood, children’s primary socialization models are their parents [1]. Through their parents’ input, toddlers can learn the sociocultural values and norms of their culture so that they will communicate and act in culturally appropriate ways [2]. For example, parents from individualistic societies tend to highlight the thoughts and beliefs of others compared to parents from collectivist societies, where independent thoughts and beliefs impede group harmony [3,4,5]. Instead, parents from collectivist societies tend to emphasize understanding others’ desires to promote intergroup harmony [4]. The language in which parents communicate the beliefs and desires of others is referred to as parental mental-state-talk.

Parental mental-state-talk affects several aspects of a child’s social development, including but not limited to sociocultural values and norms, socioemotional competence, and theory of mind reasoning, even after controlling for age and language [6,7]. To date, parental mental-state-talk has been primarily examined in monolingual English-speaking populations, and only a few studies have examined parental mental-state-talk in bilingual populations [8,9,10,11]. There, only one of the parents’ languages, English, was examined [10,11], and thus research on parental mental-state-talk has yet to capture bilingual parents’ mental-state-talk in its entirety. If parents use language to transmit sociocultural values and norms, then differences in bilingual parents’ mental-state-talk might arise where their two languages differ in sociocultural values and norms. The main aim of the current study was to examine if mental state expressions differ across languages in bilingual mothers. 

In daily interactions, parents often highlight the thoughts (‘’cognitive’’ mental-state-talk) and wishes (‘’desire’’ mental-state-talk) of others through their mental-state-talk. Mothers frequently talk about both desires and cognitions in child-directed speech [12,13,14,15,16,17]. Frequencies of and transitions between talking about desires and talking about cognitions may in part reflect mothers adjusting their speech to be suitable for their child’s age and competence [13,15,16,17,18,19], as children typically acquire words to refer to desires first and later acquire words to refer to cognitions [12,20]. 

Across cultures, one prominent difference in parental mental-state-talk is the frequency of cognitive mental-state-talk noted between English-speaking and Chinese-speaking parents [8,11,21,22,23,24]. This language difference is attributable to cultural differences, where English-speaking parents, typically valuing independence, are more likely to describe and explain an individual’s thoughts [5,25,26], and Chinese-speaking parents, typically valuing interdependence, are more likely to focus on social norms [25,27,28] and understanding the needs and desires of others [4]. Thus, the English-speaking culture focuses on thoughts whereas the Chinese-speaking culture focuses on one’s behaviors and others’ desires. 

Some studies have examined differences in Chinese-speaking and English-speaking parents’ mental-state-talk [8,10], but little can be concluded as to why a difference emerged between the cognitive mental-state-talk of these two groups of parents. This is because researchers have only compared maternal mental-state-talk in Chinese-speaking parents residing in Eastern, interdependent societies and English-speaking parents residing in Western, independent societies. Unlike studying two monolinguals, studying bilinguals allows researchers to examine cross-linguistic differences within the same individuals. However, some caution must be heeded as studying bilinguals is not equivalent to studying two languages separately in two monolinguals [29,30,31]. In the present study, we take an initial step in investigating language-specific (or general) effects of maternal mental-state-talk in English–Mandarin bilingual mothers on their toddlers residing in a multilingual and multicultural society: Singapore.

Singapore is a diverse society that prides itself in striking a balance between adopting modern Western values while retaining traditional Eastern values [32,33,34]. A prominent example of how the Singaporean government strikes this East–West balance is through encouraging citizens to maintain at least two languages. From an early age, Singaporean children learn both English and their ‘’mother tongue’’ in school. In Singapore, English is used as the common working language to unite communication among all Singaporeans and is the lingua franca for both business and instruction. By contrast, Singaporeans’ mother tongues (Mandarin for Chinese Singaporeans, Malay for Malay Singaporeans, and Tamil for Indian Singaporeans) are used to promote a sense of cultural belonging and values. As such, mother tongues are primarily used at home and within one’s ethnic community. By dividing the functions of English and mother tongue languages, the Singaporean government has attached different social goals and functions to each language. Such a division of social goals and functions is referred to as “diglossia” and is argued to lead to bilingualism that perseveres and endures across generational changes [31]. In addition, associating different social goals and functions with different languages may be reflected in Singaporean maternal mental-state-talk as Singaporean mothers may promote individualistic thinking and norms in English but collectivist thinking and norms in their mother-tongue (Mandarin) as previous research has shown the impact of cultural values in caregivers’ mental-state-talk [11]. 

For the present study, we explored how bilingual mental-state-talk may differ between two languages. To elicit maternal mental-state-talk in bilinguals, mothers were asked to engage in two free-play sessions, where they were instructed to exclusively use English for one of the sessions and Mandarin for the other session. Free-play sessions were used over story-telling sessions for three reasons. First, meta-analysis indicates that there are no quantitative differences in maternal mental state references between story-telling and naturalistic interactions [6]. Second, free-play is more likely to provide a representative sample of mothers’ and children’s daily interactions compared to storytelling. Third, the study’s focal interest is ad lib cognitive and desire mental-state-talk, which are more likely to occur in free-play than in constrained story-telling contexts [35].

We had two predictions. First, we expected to replicate previous findings showing that mothers frequently use desire mental-state-talk when engaging with their 18-month-old toddlers. Thus, regardless of language, mothers will produce more expressions of desire than expressions of cognition. Second, we expected the frequency of mental-state-talk to differ depending on the language mothers spoke. Specifically, because English is promoted as the lingua franca for business and education, and mother tongues are used as the language for home and in-group interactions in Singapore, mothers should produce more cognitive expressions in English than in Mandarin and more desire expressions in Mandarin than in English, reflecting each language’s functions and roles in Singaporean society.

## 2. Materials and Methods

Thirty Singaporean-Chinese English and Mandarin bilingual mothers (*M* = 33 years, *SD* = 3 years, range = 29 years to 40 years) and their toddlers (16 females, *M* = 19.3 months, *SD* = 2.1 months, age range = 15.8 months to 24.8 months) participated. Mothers were recruited online through Facebook pages and were asked several screening questions, including whether the mother would be able to spend 30 min alone with her toddler and whether the mother would be comfortable with speaking exclusively English and speaking exclusively Mandarin for 15 min each. Mothers had either a university degree (70%), a post-graduate degree (10%), a junior college/polytechnic diploma (13%), or completed secondary school (7%). Most mothers reported that they were currently employed (77%). The remaining mothers reported that they were on leave from work (3%), currently unemployed (7%), or a homemaker (13%). Most mothers reported that their child’s preferred language was English (73%), while the rest either reported that their child’s preferred language was Mandarin (20%) or had no preference (7%). Toddlers’ average daily percentages for hearing each language were also collected. Two mothers reported percentages that summed to greater than 100%. Excluding these mothers, mothers reported that their toddlers heard more English on average (*M* = 67%, *SD* = 23%) than Mandarin (*M* = 34%, *SD* = 22%). Including the two mothers whose summed percentages exceeded 100% produced a similar result (English: *M* = 66%, *SD* = 21%, Mandarin: *M* = 35%, *SD* = 21%). Mothers were compensated with a small monetary amount, and toddlers received a certificate for their participation. The study was approved by the Institutional Review Board of Nanyang Technological University (NTU-IRB-2014-11-010). All data have been made available at the following URL: https://doi.org/10.21979/N9/1KTUHC.

Mothers were provided with a standard set of toys to use in the free-play session. These toys included a doll, a blanket, a tea set (including a tea pot with a lid, two cups, two saucers, and two spoons), a toy cellphone, a train, a foam ball, five nesting barrels, and two storybooks, ‘’Guess How Much I Love You’’ and ‘’The Very Hungry Caterpillar’’. The language of the storybooks matched the language of the free-play session (i.e., the English versions were provided for the English free-play session and the Mandarin versions were provided for the Mandarin free-play session). 

Mothers first completed a demographic questionnaire and two vocabulary checklists, one in English and one in Mandarin. Specifically, the short-form versions of the MacArthur–Bates Communicative Development Inventory [36] and the Mandarin Communicative Development Inventory [37] were used to measure toddlers’ bilingual expressive vocabulary. Mothers were instructed to mark down the words they have heard their child produce. Total vocabulary size in each language was then tabulated by summing the number of ticked words in the list.

They were then asked to engage their toddler in two 10 min free-play sessions, where they were asked to speak exclusively in English for one session and exclusively in Mandarin for the other session. Language order for the two free-play sessions was counterbalanced across participants. Free-play sessions were video-recorded. 

Speech from the two free-play sessions was transcribed by one of four English–Mandarin bilingual research assistants using the Computerized Language Analysis (CLAN) program [38]. Each transcript was reviewed by at least one other research assistant, and any discrepancies in the transcripts were resolved through discussion. Standard orthography was used for the English transcripts, and Mandarin characters were used to transcribe the Mandarin transcripts.

Mental state words were taken from existing studies on maternal mental-state-talk [15,32] and were categorized as either cognitive or desire mental-state-talk (Table 1). Cognitive words referred to any kind of thought process, such as “understand/了解” or “believe/相信”. Desire words referred to a wish and were expressed in words such as “want/要” and “hope/希望”. Mandarin mental state words were adjusted to those that are more commonly used by Singaporeans. The ‘’kwal’’ command in CLAN was used to extract all utterances that contained a mental state word. Because of the age of the toddlers in the current sample, few produced mental state words. Thus, only mothers’ mental-state-talk was coded. 

Mothers infrequently code-switched between languages (i.e., using English in the Mandarin-designated session and Mandarin in the English-designated session). Including English types and Mandarin types outside of their designated language session, mothers produced less than 5% of English types in the Mandarin-designated session and less than 1% of Mandarin types in the English-designated session. In total, there were eight occurrences of mental-state-talk outside of their designated language session. There were three occurrences of “要” (“want”) across English sessions and five occurrences of “want” across Mandarin sessions. 

To capture maternal mental-state-talk, researchers have calculated either a sum to measure absolute frequency of mental-state-talk [15] or a proportion to control for speaker verbosity [40,41]. However, a meta-analysis has revealed that mental-state-talk frequency is more sensitive as a predictor for later child outcomes than proportion of mental-state-talk [6]. Thus, we calculated maternal mental-state-talk as a frequency variable. Only words that reflected a state of mind were extracted and tabulated towards the mental state word frequency count. Additionally, mental state words from fixed phrases or phrases from books (e.g., “He wanted to be sure that Big Nutbrown Hare was listening” from ‘’Guess How Much I Love You’’), repetitions within three utterances, and words with ambiguous meanings were excluded from the total tabulation. 

English and Mandarin mental state words were first coded by two independent coders. A third coder coded both sets of transcripts. All coders reviewed all extracted words with the transcript and the accompanying video to examine how each targeted word was used. Cohen’s kappas, 0.87 for English and 0.73 for Mandarin, indicated high interrater reliability for both English (92% agreement) and Mandarin (84% agreement) mental state word coding. The following analyses used the first coders’ mental state frequency counts. Mental state type was calculated as frequency for each mental state word category.

## 3. Results

Overall, mothers produced a total of 11,725 utterances (*n* = 5868 English utterances, *n* = 5857 Mandarin utterances), and their toddlers produced 2206 utterances (*n* = 1089 English utterances, *n* = 1117 Mandarin utterances). There was no difference between the numbers of utterances produced in the English sessions and the Mandarin sessions for both mothers, *t*(29) = 0.04, *p* = 0.97, *d* < 0.01, or toddlers, *t*(29) = −0.19, *p* = 0.85, *d* = 0.04. Speech richness between the two languages was also examined by conducting *t*-tests on type-token ratios. Type-token ratio was calculated by dividing the number of types produced in one language by the number of tokens produced in the same language. Higher type-token ratios indicate more complex speech. Type-token ratios did not differ between languages for either mothers, *t*(29) = −0.22, *p* = 0.83, *d* = 0.04, or toddlers, *t*(29) = 1.78, *p* = 0.08, *d* = 0.324.

Toddlers’ reported English vocabulary (*M* = 26.23, *SD* = 21.92) was larger than their reported Mandarin vocabulary (*M* = 10.56, *SD* = 14.79), *t*(29) = 4.42, *p* < 0.01, *d* = 0.81. There were no significant correlations between toddlers’ age, maternal educational level, and the frequency of cognitive and desire maternal mental-state-talk. There were significant positive relationships between toddlers’ age and their reported English vocabulary, *r*(28) = 0.75, *p* < 0.01, as well as their reported Mandarin vocabulary, *r*(28) = 0.41, *p* < 0.05.

**Figure 1:**
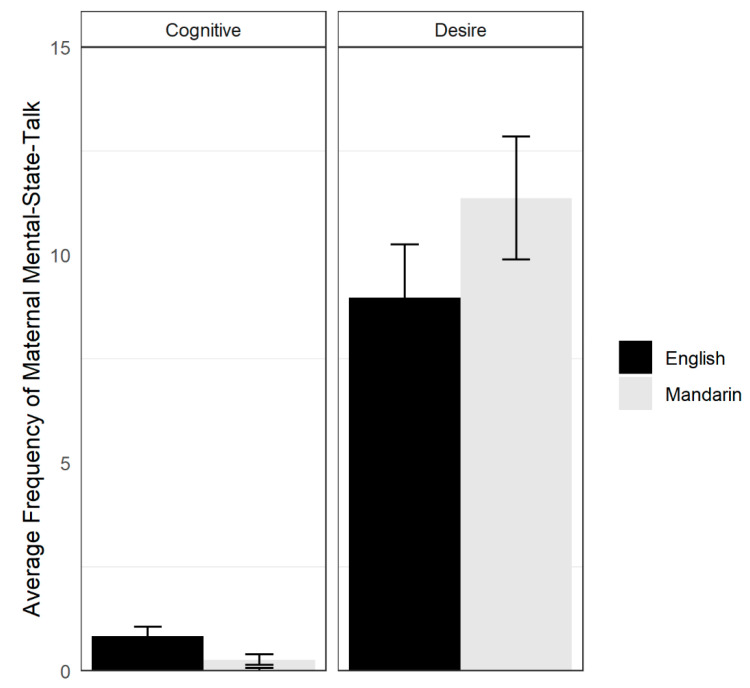
Average frequencies of maternal mental-state-talk across languages with error bars representing ±1 standard error of the mean. Average frequency of mothers’ cognitive mental-state-talk is illustrated on left; average frequency of mothers’ desire mental-state-talk is illustrated on right.

Total maternal mental-state-talk as the sum of cognitive and desire mental-state-talk for each language was calculated. Total maternal mental-state-talk did not differ between languages, *t*(29) = −0.48, *p* = 0.63, *d* = 0.15. A 2 (Language) × 2 (Mental State Type) repeated-measures ANOVA revealed a main effect of Mental State Type, *F*(1, 29) = 58.52, *p* < 0.01, η_ges_^2^ = 0.42. Overall, mothers used more desire (*M* = 10.20, *SD* = 6.85) than cognitive (*M* = 0.97, *SD* = 1.17) words. There was also an interaction between language and mental state type, *F*(1, 29) = 8.54, *p* < 0.01, η_ges_^2^ = 0.03. Follow-up pairwise *t*-test comparisons revealed that mothers used more cognitive words in English (*M* = 1.63, *SD* = 2.67) than in Mandarin (*M* = 0.30, *SD* = 0.79), *t*(29) = 2.98, *p* < 0.01, *d* = 0.54. There was also a marginal difference in the frequency of maternal desire mental-state-talk, *t*(29) = −2.01, *p* = 0.054, *d* = 0.37; mothers produced more desire words in Mandarin (*M* = 11.37, *SD* = 7.04) than in English (*M* = 9.00, *SD* = 8.09). See Figure 1.

**Figure 2:**
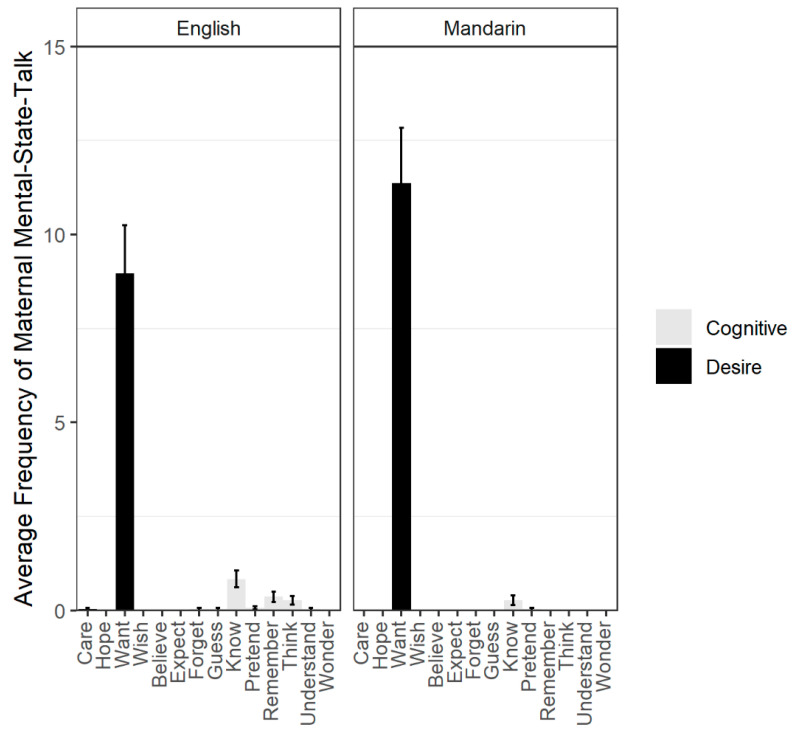
Average frequencies of maternal mental-state word across languages with error bars representing ±1 standard error of the mean. Average frequency of mothers’ English mental-state-talk is illustrated on left; average frequency of mothers’ Mandarin mental-state-talk is illustrated on right.

Upon closer examination of the frequency of mental-state words produced, we found that mothers’ desire production was dominated by the production of “want.” In fact, the only other desire word produced, “care”, was produced only once by a mother in the English session; no other desire words were produced in the Mandarin session. Overall, the cognitive mental state word, “know” (“会不会“, “会”, and “懂”) was produced most frequently in each language. For Mandarin, only one other cognitive mental state, “pretend,” was produced and only once across sessions. There was more variation in English cognitive mental state production. Aside from “know” (*n* = 25 total occurrences), mothers produced the cognitive words, “forget” (*n* = 1 occurrence), “guess” (*n* = 1 occurrence), “pretend” (*n* = 2 total occurrences), “remember” (*n* = 11 occurrences), “think” (*n* = 8 occurrences), and “understand” (*n* = 1 occurrence). The average frequency of each mental state produced by mothers is illustrated in Figure 2.

Most cognitive expressions were used to refer to the child’s cognitive state rather than the mother’s or a third person’s cognitive state. Specifically, all occurrences of “know” in Mandarin were mothers asking their toddler about their (the toddler’s) knowledge capacity. For example, one mother asked her toddler, “懂 什么 是 青色 吗” (“Know what is green?”). Similarly, in English, mothers primarily (apart from three instances of “know”) asked their toddlers about their knowledge capacity. For example, one mother asked, “does xxx know how to jump?” where “xxx” is the toddler’s name. For the other instances of “know” mothers referred to their own knowledge state (*n* = 1) and a third person’s knowledge state (*n* = 2). In most instances of “remember,” mothers were asking their toddler if the toddler could recall a previous event except for two instances where mothers used the plural first person, “we.” Similarly, “forget,” “guess,” “understand,” and “pretend” were used to refer to the child’s cognitive mental state. However, unlike other cognitive mental states, “think” was used primarily to refer to the mother’s own cognitive state (7 out of 8 occurrences).

It is possible that Singaporean mothers adjusted their speech according to their toddler’s competency [13,15,16,17,18,19] rather than associated different socialization goals and functions to each language, as most toddlers were reported to prefer English and were also reported to be English dominant. Singaporean mothers’ awareness of their child’s language competencies may result in more sophisticated maternal cognitive mental-state-talk in English and simple maternal desire mental-state-talk in Mandarin. Follow-up analyses were conducted to test the possibility that mothers adjusted their maternal mental-state-talk complexity to their child’s perceived language preference. Total cognitive mental-state-talk was computed by adding the frequency of maternal cognitive mental-state-talk. Total desire mental-state-talk was computed similarly. Two toddlers were excluded from this analysis because mothers reported that their toddler did not have a language preference. There was no difference between total maternal cognitive mental-state-talk and toddlers’ reported language preference, *U* = 42.00, *p* = 0.17. Similarly, there was no difference between total maternal desire mental-state-talk and toddlers’ reported language preference, *U* = 50, *p* = 0.39.

Mothers’ perception of their toddlers’ language dominance based on their reported vocabulary was also considered as a potential factor in mothers’ mental-state-talk frequency. Toddlers’ language dominance was determined by comparing toddlers’ reported English vocabulary and their reported Mandarin vocabulary. Toddlers with greater reported English vocabulary than reported Mandarin vocabulary were categorized as “English dominant”. Similarly, toddlers with greater reported Mandarin vocabulary than reported English vocabulary were categorized as “Mandarin dominant.” Two toddlers had the same reported vocabulary scores for both languages and were excluded from the analysis. Again, there were no differences between total maternal cognitive mental-state-talk and toddlers’ reported language dominance, *U* = 55.00, *p* = 0.90, or between total maternal desire mental-state-talk and toddlers’ reported language dominance, *U* = 44.5, *p* = 0.45.

## 4. Discussion

Mental-state-talk research has been focused on how monolingual parents’ mental-state-talk affects their children’s development [7]. However, monolingualism is becoming globally less common [42], and thus more attention should be paid to the effects of bilingual parental speech on their child’s development. We extended this line of research by investigating bilingual English–Mandarin Singaporean mothers who, due to the demands of the environment, associate different yet specific functions to each of their languages: English for business and education and Mandarin for in-group contexts. The current study is the first to explore how bilingual maternal mental-state-talk differs in two languages within the same individual.

We found that Singaporean mothers produced mostly desire expressions in both their languages, but the same mothers produced more desire expressions when speaking in Mandarin to their children and more cognitive expressions when speaking in English. Cognitive expressions were frequently used to refer to the child’s cognitive state except for “think,” which, in contrast, was primarily used to refer to the mother’s own cognitive state.

Before the child’s second birthday, monolingual mothers typically use more desire vocabulary [43]; it is not until approximately their child’s second birthday that monolingual mothers switch to using more words that refer to cognitions [12,13,15,16,17,20]. Like monolingual mothers, bilingual mothers in the present study produced more words referring to desires than cognitions regardless of the language, showing that expressions of desires in child-directed speech precede other mental state expressions even in a bilingual population.

Although mothers as a group used primarily desire mental-state-talk for both languages, bilingual English–Mandarin Singaporean mothers used more cognitive words in English than in Mandarin and more desire words in Mandarin than in English. This difference in this context suggests that Singaporean mothers may be using mental-state-talk as a vehicle to transmit the corresponding social norms for each language. Previously, Mandarin-speaking children have demonstrated a precocious use of words pertaining to desires, but a delay in using words pertaining to cognitions compared to English-speaking children [39]. This difference has been argued to reflect mothers’ disparate cultural goals in socializing children, as individualistic thinking is valued in independence-centric cultures but is disruptive for interdependence-centric cultures [8,11]. To transmit such cultural goals, mothers are argued to tailor their mental-state-talk such that mothers from interdependent cultures (e.g., Chinese) rarely mention what another individual may be thinking [39]. Indeed, the present research found that in Mandarin, when using a cognitive expression (know and pretend), mothers only referred to their child’s mental state. In contrast, for English, mothers tended to deviate from focusing on the child’s mental state when using the cognitive state, “think”.

Despite the unique contribution to understanding bilingual mothers’ mental-state-talk, the current study has some limitations and raises several questions. First, the study is cross-sectional, limiting our understanding of the development of bilingual maternal mental-state-talk and how it may later affect children’s development. Future studies could implement a longitudinal design to address this limitation and examine trends of bilingual maternal mental-state-talk. A longitudinal design would also allow researchers to examine if, and when, bilingual mothers switch between desires and cognitions, and whether transitions depend on children’s respective language proficiency. Second, although the Singaporean government assigns English as the language for international communications and commerce, highlighting independence, and mother tongues (e.g., Mandarin) as the language for cultural and heritage-related communications, highlighting interdependence [2,44], it is unclear whether Singaporean mothers actually attribute different sociocultural values and norms to English and Mandarin. Future mixed-methods research could directly interview mothers and probe their beliefs about each language’s function. Third, the findings produced by this study may have emerged for a reason apart from differences in transmission of cultural values. If Singaporeans truly associate different social goals and functions to English and their mother tongue as intended by the Singaporean government, this distinction should extend to other ethnic groups in Singapore. Future research could investigate mental-state-talk in other bilingual Singaporean parents (e.g., Malays and Indians who speak English-Malay and English-Tamil, respectively) to examine whether their speech within each language similarly reflects societal imposed functions. We would expect, if Singaporean mothers socialize their children in the respective functions of each language, then English-Malay and English-Tamil parents would also produce more cognitive words in English than in their mother tongue and produce more desire words in their mother tongue than in English as the Singaporean English-Chinese parents in the present study.

## 5. Conclusions

Extensive research has examined the significance of maternal mental-state-talk in children’s development, but with bilingualism becoming increasingly common, it is also critical to examine the characteristics of bilingual mothers’ mental-state-talk. In Singapore, bilingualism is encouraged, but each language’s functions differ. English is deemed as a common language that unites Singaporeans, and Singaporeans to Westerners, in public business and educational spaces. Singaporean mother tongues, such as Mandarin, are languages to be used at home or with an individual’s ethnic group. In the present study, bilingual English–Mandarin Singaporean mothers produced mostly words referring to desires. There were also cross-linguistic differences in mental-state-talk. Mothers used more cognitive mental state words in English than in Mandarin, and more desire mental state words in Mandarin than in English. This cross-linguistic difference cannot be explained by the mother’s perception of their child’s language preference or dominance. The government’s demarcation of social values and norms for each language may have contributed to variations in how the Singaporean mothers use mental state words in each language, and thereby, socialize children to understand the different roles that each language plays in their society.

## Figures and Tables

**Table 1:** List of English mental state words and their Mandarin translated counterparts.

	English	Mandarin
**Cognitive**	Believe	相信,信
	Expect	期望,会,会不会
	Forget	忘
	Guess	猜
	Know	知道,会,懂
	Pretend	假装,装
	Remember	记
	Think	想 ^1^
	Understand	了解,理解,明白
	Wonder	想 ^1^
**Desire**	Care	关心
	Hope	希望
	Want	要,想 ^1^
	Wish	愿望

^1^ Some Mandarin mental state words are polysemous, resulting in some mental state words appearing twice or even in different mental state categories [39]
